# Fungal Endophytes Promote Tomato Growth and Enhance Drought and Salt Tolerance

**DOI:** 10.3390/plants9070877

**Published:** 2020-07-10

**Authors:** Mustafa Morsy, Blake Cleckler, Hayden Armuelles-Millican

**Affiliations:** Department of Biological and Environmental Sciences, University of West Alabama, Livingston, AL 35470, USA; clecklerb@gmail.com (B.C.); millicanh88@gmail.com (H.A.-M.)

**Keywords:** fungal endophyte, abiotic stress, drought, salinity, *Solanum lycopersicum*

## Abstract

In a search for efficient fungal endophytes that can promote crop production and/or increase crop tolerance to abiotic stress, we isolated and tested various species harbored by wild plants. Sixty-seven endophytic fungal isolates were obtained from drought stressed, poor soil habitats, and inland high salt areas. We extensively tested the roles of *Ampelomyces* sp. and *Penicillium* sp. isolates in improving tomato growth and yield. Under greenhouse and field trails, *Ampelomyces* sp. and *Penicillium* sp. endophytes proved effective in conferring positive benefits to tomatoes placed under stress as well as under normal growing conditions. *Ampelomyces* sp. conferred tolerance to tomatoes placed under drought stress in addition to enhancing overall plant growth and fruit yield in comparison to non-symbiotic plants under drought stress. *Penicillium* sp. conferred tolerance to tomatoes placed under 300 mM salinity stress in addition to enhancing root biomass in comparison to non-symbiotic plants. Both endophytes proved efficient in enhancing plant growth, stress tolerance, recovery, and fruit yield under optimal experimental conditions in comparison to non-symbiotic plants. Field testing of tomato yield showed increased yield of symbiotic tomatoes compared to non-symbiotic ones. This data suggests that both *Ampelomyces* sp. and *Penicillium* sp. share a promising potential for improving future agricultural production, particularly with the projected changes in climate in the future.

## 1. Introduction

Most plants serve as unique ecological hosts for diverse communities of enigmatic endophytic fungi that live within plant tissues without causing any disease or obvious negative symptoms [1,2,3,4]. Endophytic fungi have been associated with plants for more than 400 million years and have been recovered from all living plants examined for their presence [5]. The communities of microbial symbionts that reside within host organisms are far more diverse than those of the host organisms [6,7], indicating the crucial roles played by these microbes in the function and survival of host plants.

Fungal endophytes, particularly non-clavicipitaceous forms, establish mutualistic relationships with plants and provide the hosts with certain benefits [8,9]. Plants carrying fungal endophytes can withstand biotic and abiotic stresses including excessive salt, drought, and heat, in addition to improving the acquisition of nutrients and increasing plant growth or fruit yields [10,11,12,13,14]. For example, *Curvularia protuberata* and its host plant *Dicanthelium lanuginosum* grow in geothermal soils at temperatures up to 65 °C. When grown non-symbiotically, neither plant nor fungus is able to survive temperatures over 40 °C [15,16]. Similarly, the fungus *Fusarium culmorum,* isolated from the coastal dune grass (*Leymus mollis*), confers salt tolerance ranging from 300–500 mM NaCl in tomato [17], while *Penicillium minioluteum* confers salinity stress resistance in soybeans (*Glycine max*) [18]. In addition, fungal endophytes can produce bioactive alkaloids that increase resistance of host plants to plant pathogens as well as to vertebrate and invertebrate herbivores [19,20,21]. For example, *Cryptosporiopsis* sp. confers disease resistance to pathogens in barley (*Hordeum vulgare*) and larch (*Larix decidua*) [22]. Moreover, *Fusarium* sp. reduces infection of *Pyrenophora tritici-repentis,* which causes tan spot in wheat (*Triticum* sp.) [23]. Furthermore, endophytic fungi can enhance fruit quality by increasing soluble sugar production in apples (*Malus domestica*) cultivar Honeycrisp [24].

Fungal endophytes have long been thought to be restricted to specific lifestyles: mutualistic, neutral, or parasitic. However, studies suggest that fungi can express one or more lifestyles depending on the host’s genetic variation or environmental factors. For example, individual isolates of pathogenic species of *Colletotrichum* can express a mutualistic lifestyle in healthy hosts, conferring growth enhancement, disease resistance, and drought tolerant [25]. Pathogenic and non-pathogenic fungi have been isolated from asymptomatic plant tissues, implying that both mutualistic and pathogenic fungi remain dormant within plants until senescence, giving endophytes access to plant nutrients as they become available [1]. The initial phases and colonization of pathogens, mutualists, and commensals are identical for diverse fungi, making lifestyle expression a post-colonization phenomenon that involves biochemical or genetic communication between host and symbiont [12]. Lifestyle switching occurs in such genetically divergent plants such as the cucurbitaceous and solanaceous families, as well as in their cultivars, e.g., tomato (*Solanum lycopersicum*) [25]. In addition, the occurrence of endophytes in natural grass populations positively correlates with water stress [26,27,28,29,30].

Fungal endophyte diversity represents 7% of the 1.5 million fungi on earth [7,31], making fungal endophytes a treasure for novel applications. Presumably, many endophyte species remain to be discovered, as well as their ecological roles in nature [1]. One of the noticeable advantages of endophytic fungi for research is their ability to grow in vitro and applicability to plant hosts under controlled experimental conditions to analyze the potential benefit to their hosts. A straightforward approach to test this is to identify novel endophytes and then compare host performance of the same genotype with and without the fungal endophyte [32]. Any difference in growth or tolerance between symbiotic and non-symbiotic plants can be accredited to the endophyte [33]. Many researchers have proposed biotechnological application of many fungal endophytes that promotes growth of a vast range of plant hosts [34] and the use of plant microbiome to improve crop yield is a promising tool to help feed the growing human population [35,36].

The objective of the current study is to discover novel fungal endophytes associated with wild plants growing in stressful habitats and to evaluate their roles in providing growth benefits and stress tolerance. Tomato (*S. lycopersicum*) plants were used as a model system to test the effects of these endophytes under normal, drought, and salt-stressed conditions in the greenhouse and limited-water supply in the field.

## 2. Results

### 2.1. Plant Identification

**Figure 1:**
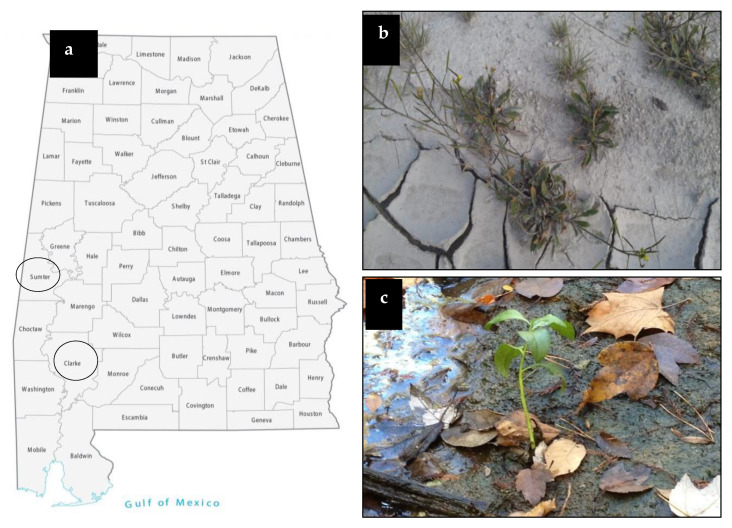
Sampling sites and represented collected plants. (**a**) Alabama map showing 2 counties used for sampling, circled. Sumter County and the Stimpson Wildlife Sanctuary of Southern Clarke County, (**b**) *Pyrrhopappus carolinianus* plant (SC8) collected from Sumter County, (**c**) *Acer negundo* plant (CC10) collected from Clarke County of soil with 712 PPT salt.

Forty different plant samples growing in harsh habitats were collected from Clarke and Sumter counties in Alabama, during the months of February and April 2012 and 2013 (Figure 1). Plants identification showed that plants collected from Sumter County belong to only 4 genera in 2 families (Plantaginaceae and Asteraceae), while plants from Clarke County belong to 9 genera in 7 families. Asteraceae were common in both locations, but were represented by different genera, suggesting that most plants identified were endemic to their habitat (Table 1 and Table 2).

Almost all collected plants harbored some fungal endophytes. The total infection rate of all explant was approximately 91%. Fungal endophytes growing from each explant were identified based on phenotypic appearance and scored (Table 1 and Table 2). Sixty-seven phenotypically different endophytes were isolated into pure cultures (33 from Sumter County plants and 34 from Clarke County plants). The number of phenotypically different endophytes per plant ranged from 1 to 5 types and the number of explants infected with each phenotype were scored (Table 1 and Table 2). All pure cultures were identified based on their internal transcribed spacer (ITS) DNA sequences with National Center for Biotechnology Information (NCBI) accession and percentage of identity listed in Table 1 and Table 2. Phenotypic and molecular identification indicated that these fungi belong to 30 fungal genera and 46 different species. Of the 67 total fungal isolates, 35 isolates identified as potential plant pathogens, 21 isolates as endophytic fungi, and 11 isolates as unknown, facultative pathogen, or soil-borne fungi.

### 2.2. Soil Analysis

Soil samples surrounding each plant were analyzed. The pH of soil samples of Sumter County plants was alkaline, ranging from 7.8 to 8.5, while the pH of Clarke County soil was between 6.4 and 7.9. The total dissolved salts (TDS) of Sumter County soils ranged from 36–55 mg mL^−1^, except for SC7 and SC8 plants, which were 89 and 107 mg mL^−1^, respectively (Table 3). The TDS of soil in Clarke County ranged from less than 15 mg mL^−1^ to over 700 mg mL^−1^ for plant CC3, with higher TDS near the salt springs (Table 3). In Clarke County, soil salinity for 5 samples was extremely high, between 403 and 712 mg mL^−1^. These samples were taken on the direct perimeter of the salt spring, including samples CC1 through CC5. Other samples were taken farther from the salt spring perimeter (CC6 at 6 m, CC7 at 10 m, and CC8, CC9, and CC10 samples at more than 20 m from the salt spring). Qualitative measurements of macronutrients (N, P, K) generally shows that all soil samples were poor with low levels of N, low/medium levels of P and high levels of K (Table 3).

### 2.3. Screening of Endophytic Fungi for Tomato Growth and Health

Eight endophytes were chosen as potential candidates for testing their role in growth promotion and/or abiotic stress tolerance. Three endophytes unique to salt habitat (*Penicillium chrysogenum*, *Chaetomium globosum*, and *Clonostachys rosea*), three endophytes unique to drought and poor nutrients (*Ampelomyces* sp., *Pilidium* sp., and *Plectosphaerella* sp.) and two endophyte isolates of *Colletotrichum gloeosporioides* obtained from drought or poor nutrient and salt-stressed habitats were used for further studies.

**Figure 2:**
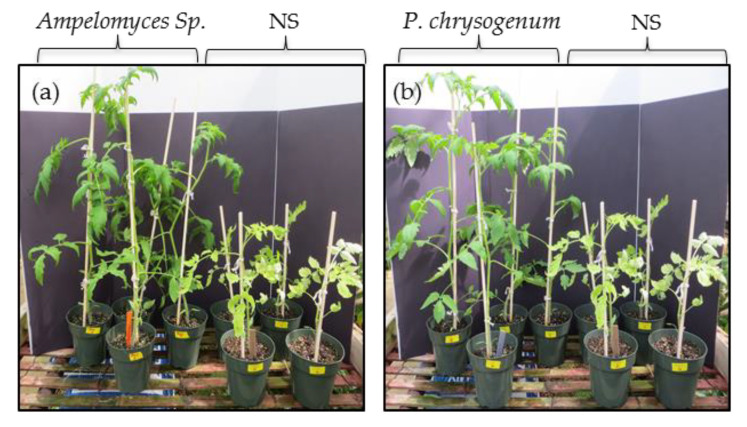
Six weeks old seedling growth comparison between non-symbiotic plants (NS) and plants colonized with fungal endophytes. (**a**) Plant colonized with *Ampelomyces* Sp. compared to NS plants. (**b**) Plant colonized with *P. chrysogenum* compared to NS plants. Both groups of colonized plants showed an increase in growth compared to NS plants.

Six-week old seedlings growing under normal conditions and colonized with *C*. *globosum*, *C*. *rosea*, *Pilidium* sp., *C*. *gloeosporioides* and *P*. *cucumerina* showed unhealthy growth including leaf color changes (yellowing and purpling), leaf curling and chlorosis (data not shown). Overall, these plants were unhealthy compared to the non-symbiotic (NS) plants, therefore, these endophytes were eliminated from the study. Remarkably, all plants colonized with *Ampelomyces* sp. or *P. chrysogenum* fungi showed very healthy and improved growth compared to NS plants (Figure 2). Therefore, we focused our study on plants colonized with *Ampelomyces* sp. or *P. chrysogenum* and eliminated other endophytes.

Plants colonized with *P. chrysogenum* were exposed to salt stress while plants colonized with *Ampelomyces* sp. were exposed to drought stress similar to that of their original habitats. Two groups of 5 NS plants served as control for both treatments.

### 2.4. Screening of Ampelomyces sp. for Tomato Drought Tolerance

**Figure 3:**
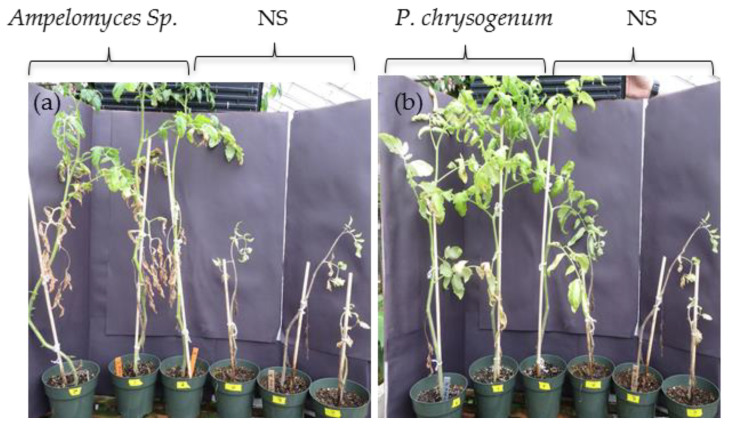
Tomato plant growth after 6 weeks of stress. (**a**) Plant colonized with *Ampelomyces* Sp. compared to NS plants, after exposure to 5 drought stress cycles. (**b**) Plants colonized with *P. chrysogenum* compared to NS plants after 300 mM NaCl applications for 6 weeks.

*Ampelomyces* sp. was isolated from *Pyrrhopappus carolinianus* plants (SC8) growing in Sumter County under drought and poor nutrient conditions (Figure 1). Plants colonized with *Ampelomyces* sp. and NS control were grown without water for 8 days before signs of wilting appeared on NS plants. Plants were watered to ¼ saturation, and left until a second wilting occurred. After 5 cycles of drought and watering that lasted 6 weeks, NS plants were either severely wilted, chlorotic, or dead. Although showing the presence of wilting and several dead leaves, plants colonized with *Ampelomyces* sp. were much healthier and survived, except for one plant (Figure 3a). Surviving plants were then transferred to 1-gallon pots and allowed to grow until fruit production under a regular watering regime with no stress.

### 2.5. Screening of P. chrysogenum for Tomato Salt Tolerance

Tomato plants colonized by the *P. chrysogenum* endophyte were isolated from box elder (*Acer negundo*) (CC2) growing in Clarke County under salt stress of 568 PPT TDS (Figure 1). Plants colonized with *P. chrysogenum* and NS plants were placed under salinity stress for 6 weeks by applying 150 mL (½ soil saturation) of 300 mM NaCl solution every 3 days. Colonized plants appeared much healthier than NS plants (Figure 3b). The first sign of salt stressing appeared in NS plants as a slight curling and desiccation of the leaves, which became more severe as the treatment progressed. Six weeks after stress, surviving plants were transferred to 1-gallon pots and allowed to grow until fruit production under the regular watering regime.

### 2.6. Plant Dry Weight of Shoots and Roots

**Figure 4:**
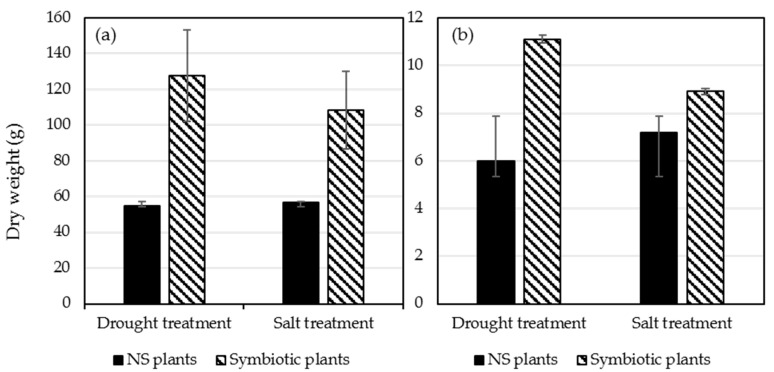
Dry weight NS plants and symbiotic plants colonized with *Ampelomyces* sp. (drought treatment) or *P.* chrysogenum (salt treatments) followed by 10 weeks without stress. (**a**) Average shoots dry weight of NS plants compared to symbiotic plants (*Ampelomyces* sp. and *P. chrysogenum*), (**b**) Average roots dry weight of NS plants compared to symbiotic plants (*Ampelomyces* sp. and *P. chrysogenum*).

Upon termination of each of the 3 experimental replicates, shoots and roots were collected and dry weight of each was calculated. Plants colonized with *Ampelomyces* sp. and exposed to drought stress showed an increase in average shoot and root dry weight compared to NS plants (Figure 4a,b). Similarly, plants colonized with *P. chrysogenum* had an increase in dry shoots and roots compared to NS plants after salt stress treatment (Figure 4a,b).

### 2.7. Greenhouse Fruit Production of Plants during Stress Recovery

**Figure 5:**
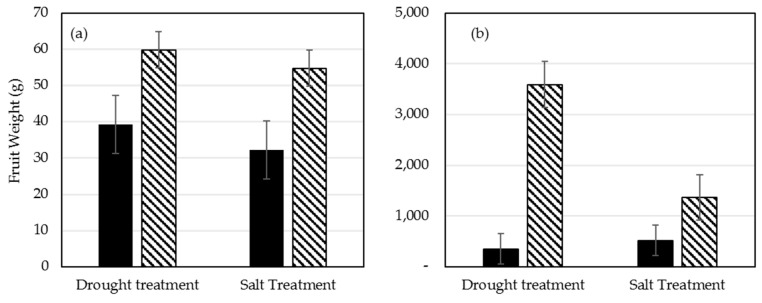
Fruit production of tomato colonized with *Ampelomyces* sp. or *P. chrysogenum* compared to non-symbiotic plants after a period of 6 weeks stress, followed by 10 weeks of normal conditions. (**a**) Average fresh fruit weight per plant of NS plants compared to symbiotic plants (*Ampelomyces* sp. and *P. chrysogenum*) (**b**) Total fruit weight produced by NS plants and plants colonized with *Ampelomyces* sp. and *P. chrysogenum*. Each data represents the means of three independent replicates and each replicate represent a minimum of 5 plants of each treatment.

After termination of drought and salt stress regimes, plants were allowed to grow under normal greenhouse conditions. Several flowers were produced in both *Ampelomyces* sp. and *P. chrysogenum* colonized plants during stress, followed by several after stress removal. NS plants were generally severely wilted and unhealthy with very few flowers. Upon the first sign of red color in the fruit, they were collected from each plant and weighed. The average fruit weight produced per plant was not significantly different between the NS plants and plants colonized with *Ampelomyces* sp. However, the number of fruit produced by each symbiotic plant was much higher than in NS plants, leading to significant increase in the total weight of fruit collected per plant colonized with *Ampelomyces* sp. (Figure 5a). Notably, plants colonized with *P. chrysogenum* had heavier fruit compared to NS plants, and the average fruit weight per symbiotic plant was higher than for NS plants (Figure 5b). In addition, the effect of salt stress on tomato production of NS plants was less severe than that of drought stress.

### 2.8. Production under Field Conditions

**Figure 6:**
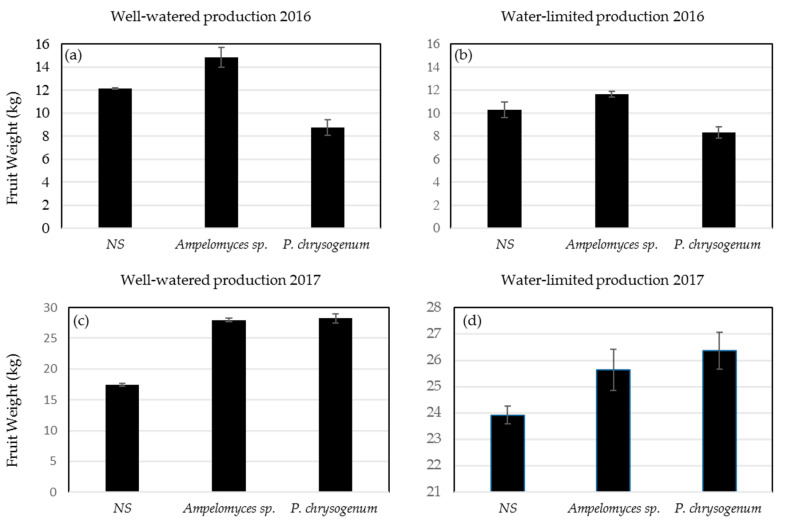
Average fruit yield of tomato plants harboring *Ampelomyces* sp. or *P. chrysogenum* and non-symbiotic plants growing in the field under two water regimes. (**a**) Well-watered conditions in 2016. (**b**) Water-limited conditions in 2016. (**c**) Well-watered conditions in 2017. (**d**) Water-limited conditions in 2017.

During the 2016 and 2017 growing season, *Ampelomyces* sp. and *P. chrysogenum* were colonized in tomato plants, grown in field conditions and compared to NS tomato control treatments under well-watered and water-limited conditions. Significant differences in yield were observed in both years between NS and symbiotic plants (Figure 6). Under well-watered conditions, plants colonized with *Ampelomyces* sp. outperformed NS plants in both years, while plants colonized with *P. chrysogenum* showed much lower yield in 2016, but higher yield when compared to NS in 2017 (Figure 6a,c). Under water-limited conditions, *Ampelomyces* sp. colonized plants yielded more fruit compared to NS plants in both years, while *P. chrysogenum* colonized plants outperformed the NS in 2017 trail only (Figure 6b,d). In 2016 and 2017, precipitation accumulation average during the growing season from April to mid-August was 1066 mm and 889 mm and an average high temperature of 30.3 °C and 29.6 °C, respectively.

## 3. Discussion

We surveyed 40 wild plants growing under stressful habitats in Alabama, and isolated fungal endophytes associated with these plants. Based on plant identification, the Clarke County samples were more diverse than the Sumter County samples; nine plant genera were identified in Clarke County and only four genera in Sumter County (Table 1 and Table 2). *Antennaria* sp. was common in both habitats. All surveyed plants contained at least one fungal species, the maximum being 5 fungal species (Table 1 and Table 2). These are expected results, as microbial symbionts have been associated with almost all plants growing under normal or stressful conditions, and in many cases plant species harbor hundreds of endophytes [1]. The low numbers of endophytes recovered per plant here was likely due to the stringent surface sterilization technique. However, the lower number of endophytes allowed isolation of only class 2 fungal endophytes abundant within plant tissue, which can be grown in vitro [5,12,22].

Analyses of all soil samples from plants collected from Sumter County showed variable soil pH and TDS, and poor soil nutrients in agreement with the soil surveys conducted in these areas by the USDA [37]. In addition, visible drought conditions surrounding some of the collected plants were observed (Figure 1a). Using data recorded by the weather station located within Sumter County and recorded by Natural Resources Conservation Service, we determined that Sumter County area had only 66 mm of rain during the collection months compared to 251 mm per month on average the same year, and an average annual precipitation of 116 mm per month [38]. These data and observations strongly indicate that the collected plants were under drought stress and/or poor nutrient conditions. Similarly, soil analyses of plants collected from Clarke County (CC-1 through CC-5) have TDS ranging from 15 to 712 PPT. The high salt levels in many areas of Clarke County, particularly in the Stimpson Wildlife Sanctuary, are well known [39].

The most common fungal genus isolated from Clarke County was *Fusarium* sp., which was present in both plants growing at high salt stress or near fresh water. *Fusarium* sp. was also isolated once from drought/poor nutrient soil of Sumter County (Table 1 and Table 2). *Fusarium* sp. has been isolated from diverse environments, and most of its species are pathogenic, though some isolates can confer salt tolerance [17]. Therefore, we rejected *Fusarium* sp. as a candidate for further testing. Endophytes from the *Colletotrichum* genera were the most common endophytes isolated from plants growing in Sumter County under drought/poor nutrient soil, with equal frequency in Clarke County. *Colletotrichum* sp. has been isolated in several studies from diverse habitats, with some aggressively pathogenic species such as *Colletotrichum gloeosporioides* [40]. However, we chose two isolates of *C. gloeosporioides*, one from each habitat, as candidates for further testing. *Penicillium chrysogenum*, *Chaetomium globosum*, and *Clonostachys rosea* were unique to salt-stressed habitats and have exhibited potential application as biocontrol agent to increase salt and H_2_O_2_ tolerance [41,42,43], therefore we chose them for further testing. Three endophytes, *Ampelomyces* sp., *Pilidium* sp., and *Plectosphaerella* sp., were unique to plants collected from drought and poor nutrient environment. We chose these three endophytes for further testing as their reported effects on plants are diverse. *Ampelomyces* sp. is described as a fungal endophyte whose most common species is *A. quisqualis,* which is commercially used as a mycofungicide to control powdery mildew in cucumbers, carrots and mangoes [44,45,46,47]. *Pilidium* sp. has been reported as a plant pathogen [48], while *P. cucumerina* is reported to colonize Arabidopsis asymptomatically [49]. We chose some potentially disease-causing endophytes to test in tomatoes because many endophytes can switch between the pathogenic and non-pathogenic lifestyles based on the hosts or the environment [50,51,52,53]. Changes of endophyte lifestyle were observed when potentially disease-causing endophytes that showed no apparent disease symptoms in the wild plants showed disease symptoms when tested in tomato under control conditions. Six of the eight tested endophytes negatively affected tomato plant health, including leaf yellowing, necrosis, and curly leaves under greenhouse conditions. In addition, due to their ability to change their lifestyle from non-pathogenic to pathogenic, some fungal endophytes have been reported as latent fungal pathogens [54].

Remarkably, six-week-old tomato plants colonized with *Ampelomyces* sp. and *P. chrysogenum* showed a significant growth advantage compared to NS control plants under optimal greenhouse conditions (Figure 4). Additionally, *Ampelomyces* sp. and *P. chrysogenum* showed increased tomato drought and salt stress tolerance, respectively, and increased the overall fruit production after stress removal. Tomato plants colonized with *Ampelomyces* sp. isolated from plants growing in nutrient-poor soil under drought conditions were much healthier compared to NS plants under standard greenhouse conditions (Figure 2a). In addition, after 5 drought cycles in 3 weeks, plants colonized with *Ampelomyces* sp. showed significant resistance to drought compared to NS plants (Figure 3a). Most of the NS plants died due to drought, while the *Ampelomyces* sp. symbiotic plants had excellent recovery during the normal 10-week conditions following the stressed period. Plants colonized with *Ampelomyces* sp. showed significant increases in dry shoots and roots compared to NS plants (3-fold increase in shoots and about 2-fold increase in roots) (Figure 4a,b). The strong root and shoot systems, and the drought tolerance of symbiotic plants, led to fruit production with a 5-fold increase compared to NS plants (Figure 5a,b). Furthermore, the number of surviving symbiotic plants was higher than NS plants. Many studies conducted on *Ampelomyces* sp. have focused on its role as a biological control of powdery mildews on crops, while no previous studies have tested its role in plant growth or stress tolerance. For example, *Ampelomyces* sp. was found to produce major active volatile compounds that elicit systemic resistance against the pathogen *Pseudomonas syringae* [55]. For the first time, the present study has shown a positive effect of *Ampelomyces* on tomato growth and drought stress tolerance. Improvements in plant growth and drought tolerance contributed by *Ampelomyces* sp. may relate to overall enhancement of plant health and resistance to pathogens. Berg [56] suggested that the fungal genus *Ampelomyces* sp. is one of several understudied genera that could be a good model organism to demonstrate influence on plant health.

Several studies have isolated *Penicillium* sp. from wild plants growing under salt stress and found them producing gibberellin and other bioactive compounds that promote growth, grain yield and shoot biomass of various plants [57,58,59,60]. In our study, tomato plants colonized with *P. chrysogenum* showed increased salt tolerance compared to NS plants after application of 300 mM salt stress for 6 weeks (Figure 5b). Similarity, maize colonized by *P. chrysogenum* isolated from the medicinal herb *Asclepias sinaica* showed increased root weight compared to NS plants when tested for root growth [61]. In our study, plants colonized with *P. chrysogenum* showed significant increase in shoots growth compared to NS plants at normal growth conditions (Figure 2b) and under salt stress followed by 10 weeks of normal conditions (Figure 3b). Additionally, the same colonized plants showed an increase in the dry root weight compared to NS plants. (Figure 4b). Salt stress adversely affects NS plants, while *P. chrysogenum* symbiotic plants show salt tolerance and continue to grow more healthily. At the end of the experiments, the average fruit weight of symbiotic *P. chrysogenum* was much higher than that of NS plants (Figure 5b). The increased average fruit weight of symbiotic plants and higher rate of survival led to about a 4-fold increase in total fruit under salt treatment (Figure 5b). Under field condition, plants colonized with *P. chrysogenum* performed better than NS plants only during 2017 testing, under both well-watered and water-limited watering (Figure 6c,d). *Penicillium* sp. has reported to improve growth of ABA-deficient tomato under salinity stress [62], increase gibberellin production in soybean [63], has remarkable activity to solubilize insoluble mineral salts in rocks including phosphates, zinc and potassium [64,65], and has biological control ability against fungal pathogen [66]. Therefore, we speculate that *P. chrysogenum* increased tomato growth via one or more of these mechanisms, and possibly other factors.

In the current study, we report improved drought and salt stress tolerance and overall tomato growth in response to colonization with *Ampelomyces* sp. and *P. chrysogenum*. However, the mechanisms by which *Ampelomyces* sp. and *P. chrysogenum* function in tomato are completely unknown, as the mechanism governing growth promotion or stress tolerance is not within the scope of this work. Consequently, further studies are required to determine the stress tolerance and growth promotion mechanisms in response to *Ampelomyces* sp. and *P. chrysogenum* colonization. Many fungal endophytes demonstrated improvement in stress tolerance, survival, and higher yield of various crop plants compared to NS plants [11,60,67,68,69]. Potential mechanisms of fungus-mediated vitality include changes in gene expression of stress-related genes in fungal and/or plants, elicitation of stress hormones, accumulation of various osmolytes, and/or production of antioxidant enzymes [70,71,72,73,74,75,76,77,78] We speculate that the mechanisms controlling the growth promotion reported here might be due to synergetic effects of some of the previously reported mechanisms. Further studies are needed to determine the mechanisms by which these fungi promote plant growth and stress tolerance.

## 4. Materials and Methods

### 4.1. Site Descriptions and Plant Collection

We conducted surveys of plants growing in poor soils in environmentally diverse areas in Alabama that include the following. (1) Sumter County is located within the Alabama Black Belt, a crescent-shaped region that extends from northeastern Mississippi across south-central Alabama. The soil of the Black Belt is chalk-based and poor in nutrients in some areas, exhibiting mostly alkaline pH in the subsoil and acidic pH on the topsoil. Twenty individual plants were collected from this area. (2) Stimpson Wildlife Sanctuary of southern Clarke County has many salt springs located in the lower Tombigbee River drainage of the East Gulf Coastal Plain. This is a unique inland saline ecosystem located more than 120 miles from the Gulf of Mexico, with salinity ranging from 700 PPT at the salt springs to essentially 0 PPT in nearby freshwater springs and creeks. Twenty individual plants (with at least 2 morphologically similar plants) were collected from soil with gradient salt levels in southern Clarke County. All plants looked healthy and disease-free based on their phenotype, regardless of habitat, poor soil, drought, and high salt levels. Each specimen was photographed, assigned an identification number, and bagged in Ziploc^®^ bags for further laboratory analyses.

### 4.2. Isolation of Endophytic Fungi

Fungal endophytes were isolated from healthy plants. The upper root and lower stem of each plant were cut into ten 2–3 cm pieces and surface-sterilized according to Schultz [3]. Plant pieces were placed on 0.1X Potato Dextrose Agar (PDA) containing ampicillin, kanamycin, and streptomycin at 50 µg mL^−1^ of each, and incubated at 25 °C. The emerging fungal colonies were scored, and the dominant fungal endophytes represented (>80%) were subcultured into 0.1X PDA to obtain pure cultures. All pure culture isolates were grouped based on the following morphological traits: shape of the mycelium, texture of the mycelium surface, color of the fungi, production of pigments, and their diffusion into the medium and microscopic features of the spores using Illustrated Genera of Imperfect Fungi [79].

### 4.3. Plant and Fungal Molecular Identification

All the plant samples were identified using the Inaturalist application (www.inaturalist.org) and by chloroplast tRNA gene sequencing. The chloroplast tRNA gene of each plant was amplified using a Phire Plant Direct PCR Kit (Fisher Scientific, Pittsburgh, PA, USA) with universal primers according to Taberlet and colleagues [80]. While pure cultures of isolated fungi were identified by sequencing of the internal transcribed spacer (ITS) of r-RNA 5.8S region according to Gardes and colleagues [81]. PCR products were purified with a Prime GelElute Extraction Kit (Prime, Gaithersburg, MD, USA), and sequenced by DeWalch Life Technologies (Houston, TX, USA) using an ABI 3700 automated DNA sequencer. The resulting DNA sequences were identified using the BLASTN tool of the NCBI nucleotide collection (nr/nt) database.

### 4.4. Soil Analysis

For each plant collected, three topsoil samples were collected from around the plant, dried on a 65 °C oven for 2 days, and then analyzed. The soil total dissolved substance (TDS) was measured by resuspending 1 g of dried soil in 40 mL of deionized water, followed by vigorous vortexing, spun down for 3 min at 1000 rpm, after which the clear supernatant was transferred into a clean tube and conductivity was measured using an Orion Star A215 pH/Conductivity Meter displaying the TDS in PPT. Soil nitrogen (N), phosphorus (P) and potassium (K) were qualitatively measured using the LaMotte Soil Test Kit Nutrients (Chestertown, MD, USA) following the manufacturer’s recommended protocol. Nitrogen data were recorded as low, medium, and high with level ranges of 0–0.3, 0.31–6.7, and >0.67 g m^−2^ (0–30, 30–60, and >60 lbs/acre), respectively. Phosphorus data were recorded as low, medium, and high with level ranges of 0–5, 6–11, and >11 g m^−2^ (0–50, 50–100, and >100 lbs/acre), respectively. The potassium levels were presented as low, medium, and high with level ranges of 0–13.5, 14–22.5 and >23 g m^−2^ (0–120, 120–200, and >200 lbs/acre), respectively. All soil tests were performed 3 times for each soil sample.

### 4.5. Tomato Colonization and Greenhouse Testing

Tomato (*S. lycopersicum* var. Better Boy) seeds were surface-sterilized in 1.0% (*v/v*) sodium hypochlorite for 15 min with moderate agitation and rinsed 5 times with 20 volumes of sterile distilled water. Tomato seeds were germinated on sterilized vermiculite and maintained at 25 °C with a 12-h fluorescent light regime. Fifteen two-week-old, endophyte-free tomato plants were inoculated with one of eight endophytes to test their effects on tomato plants, particularly growth and stress tolerance. Fifteen non-symbiotic tomato plants (NS) were used as a negative control. Tomato seedlings were gently removed from the vermiculite media; roots were washed with sterilized water and placed in a 50-mL sterile beaker with inoculation solution containing 0.03% Agarose plus 1x Murashige and Skoog media and 10^5^ spores 50 mL^−2^ of each fungal endophyte. Tomato seedlings were incubated in the inoculation solution at 25 °C for 2 days under 12-h fluorescent light. Five plants of each treatment were surface-sterilized, as mentioned above, and checked for colonization efficiency by planting them in 0.1X PDA plates. The rest of the plants (10 each) were transferred into 6” pots filled with autoclaved 3B soil mixture and kept under greenhouse conditions (27 °C with 16-h light) for the remainder of the experiment. Plants were bottom-watered for 6 weeks to allow them to become established, and to be checked for any visual disease symptoms.

### 4.6. Abiotic Stress Assays

Drought was applied to plants colonized with *Ampelomyces* sp. and NS plants by termination of bottom-watering and allowing the soils to dry. A SMR101A Data Logger (MadgeTech, Inc., Warner, NH, USA) was used to check the soil moisture content to ensure that soil moisture levels were equivalent between both treatments. Upon plants showing wilting symptoms (severe wilting for NS, and mild for wilting for plants colonized with *Ampelomyces* sp.), each plant was rehydrated by adding 75 mL sterile water (¼ of the water needed for soil saturation). The drought regime process was repeated 5 times within 3 weeks’ period. Plants were allowed to grow for the remainder of the study (10 weeks) under normal bottom-watering until fruit were produced and collected, and average fruit size and yield were measured. Plant health was assessed and photographed weekly.

Salinity stress was applied to plants colonized with *P. chrysogenum* and NS plants by top-watering of 8-week-old plants with 150 mL (½ saturation) of 300 mM NaCl solution. The plants’ appearance and health were assessed daily and photographed weekly. Plants were allowed to grow for the remainder of the study (10 more weeks) under 300 mM salt stress until fruit were produced and collected, and average fruit size and yield were measured.

At the end of the experiment, roots and shoots were collected from all plants, stored in Ziploc^®^ bags, and placed at −80 °C. Samples were dried on a 65 °C oven for one week, then dry weight of the shoot and root systems was measured.

### 4.7. Field Planting, Location, and Climatic Conditions

Tomato plants (*S. lycopersicum* var. Better Boy) were sown on mid-March 2016 and 2017, and then colonized with *Ampelomyces* sp. and *P. chrysogenum* as mentioned above. All plants were maintained until they reached a height of about 20 cm then planted in the field. Field trials were established by early April 2016 and 2017 at the experimental station at the University of West Alabama (32°36′29.8″ N 88°11′50.7″ W). The experimental layout in both years was a randomized complete block design with three plots. For each experiment plot, 12 and 30 plants for each treatment were used in 2016 and 2017, respectively. Two field experiments were conducted each year (well-watered field and water-limited). In the well-watered experiment, a drip irrigation system was established to supply each plant with 2 L of water daily. The water-limited field was watered as needed during the first 2 weeks after transfer to the field and then it was dependent only on the rainfall. In all plots, plants were grown in three single rows with a 4 m distance between rows and a 0.5 m distance between plants in the same row. Weeds were removed by hoeing. Yield was determined every week by harvesting mature healthy fruit. The average fruit yield of the three plots of each experiment was calculated.

### 4.8. Statistical Analysis

All assays described above were repeated three times. Means and standard deviations were calculated for each independent experiment. Statistical analyses were performed by means of Student’s *t*-test using Sigma Plot 12 program, and differences were considered to be significant at *p* < 0.05.

## 5. Conclusions

To meet current and future demand of food for a rapidly growing population, novel and sustainable agricultural systems are needed to effectively use land and water resources. This is particularly important with the current climate change challenges where abiotic stress is a limiting factor to agronomic production. We have discovered several fungal endophytes harbored by plants growing under drought and stress. Two fungal strains, *Ampelomyces* sp. and *P. chrysogenum*, improved plant growth. Endophyte inoculation of tomato plants enhanced growth and yield under optimal growth conditions and under drought or salt stress conditions. The findings of the current study could have vital implications for the agricultural sector if used as biofertilizer. These findings represent a promising environmentally friendly agricultural application to mitigate the effects of climatic change on crop productivity.

## Figures and Tables

**Table 1:** Plants collected from Sumter County and their fungal endophytes. Plants were identified based on their chloroplast tRNA sequence, while isolated fungi were identified based on their ITS sequences. Fungal identity and GenBank accession number are shown along with the number of explants infected.

Plant Code	Scientific Name	Family	Identity (%)	Infected Explants	Isolated Fungi	Accession No.	Identity
SC1	*Plantago lanceolata*	Plantaginaceae	96	6	*Glomerella cingulata*	JX844157.1	100
				5	*Colletotrichum gloeosporioides*	AY266378.1	99.5
				5	*Gibberella avenacea*	GU934531.1	99
				5	*Leptosphaeria* sp.	KJ173535.1	99.73
SC2	*Plantago lanceolata*	Plantaginaceae	99	7	*Pilidium* sp.	KF367478.1	98.93
				6	*Leptosphaerulina chartarum*	GQ254687.1	98.37
				5	*Pyrenochaeta* sp.	KJ207418.1	93.52
SC3	*Solidago canadensis*	Asteraceae	99	4	*Pilidium* sp.	KF367478.1	98.29
				3	*Neopestalotiopsis mesopotamica*	KM199361.1	99.05
				2	*Plectosphaerella* sp.	DQ993622.1	94
SC4	*Solidago canadensis*	Asteraceae	98	6	*Colletotrichum gloeosporioides*	AY266378.1	99.44
				5	*Neosartorya fischeri*	LC011422.1	99.26
				5	*Plectosphaerella* sp.	DQ993622.1	97.87
				3	*Nigrospora sphaerica*	MT576586.1	100
SC5	*Antennaria neglecta*	*Asteraceae*	100	6	*Pestalotiopsis clavispora*	KM402033.1	100
				5	*Nigrospora* sp.	KF128850.1	99.82
SC6	*Antennaria neglecta*	Asteraceae	99	7	*Purpureocillium lilacinum*	KC157755.1	99.8
				6	*Pestalotiopsis* sp.	JX436803.1	98.75
				6	*Phoma* sp.	KY484799.1	98.05
				5	*Plectosphaerella* sp.	DQ993622.1	98.9
SC7	*Plantago lanceolata*	Plantaginaceae	98	7	*Colletotrichum gloeosporioides*	AY266378.1	99.8
				7	*Polyporales* sp.	JQ312208.1	99.28
				7	*Purpureocillium lilacinum*	KP068975.1	98.78
				6	*Pestalotiopsis clavispora*	EU030329.1	100
SC8	*Pyrrhopappus carolinianus*	Asteraceae	99	14	*Ampelomyces* sp.	AY513943.1	100
				2	*Alternaria* sp.	MH029119.1	99.8
SC9	*Plantago lanceolata*	Plantaginaceae	97	6	*Stagonospora* sp.	KF800186.1	96.37
				6	*Trichoderma harzianum*	KJ000326.1	99.8
				6	*Zopfiella longicaudata*	KY316385.1	99.13
SC10	*Solidago canadensis*	*Asteraceae*	96	8	*Plectosphaerella* sp.	DQ993622.1	100
				5	*Sordariomycetes* sp.	JX244023.1	100
				4	*Pestalotiopsis mangiferae*	KX778664.1	99.27
				2	*Fusarium solani*	JN983014.1	100

**Table 2:** Plants collected from Clarke County and their fungal endophytes. Plants were identified based on their chloroplast tRNA sequence, while isolated fungi were identified based on their ITS sequences. Fungal identity and GenBank accession number are shown along with the number of explant infected.

Plant Code	Scientific Name	Family	Identity (%)	Infected Explants	Isolated Fungi	Accession No.	Identity (%)
CC1	*Celtis laevigata*	Cannabaceae	99	4	*Bionectria ochroleuca*	GU934503.1	99
				4	*Fusarium acuminatum*	JQ693398.1	100
				3	*Ceratobasidium* sp.	DQ102399.1	100
				3	*Alternaria alternata*	KF881759.1	100
				3	*Aspergillus terreus*	JX863370.1	97.45
CC2	*Acer negundo*	Aceraceae	99	12	*Penicillium chrysogenum*	KP068959.1	100
				6	*Penicillium glabrum*	JQ863239.1	99
				3	*Fusarium solani*	EU029589.1	99
CC3	*Halesia diptera*	Styracaceae	99	7	*Clonostachys rosea*	KM519669.1	96
				4	*Ceratobasidium* sp.	JN648710.1	99
				4	*Fusarium avenaceum*	KF010838.1	99
				2	*Fusarium* sp.	JX914477.1	99
CC4	*Cerastium glomeratum*	Caryophyllaceae	99	6	*Ceratobasidium* sp.	DQ102399.1	100
				6	*Cladosporium cladosporioides*	KC692219.1	99
				4	*Fusarium equiseti*	KP068925.1	99
CC5	*Acer negundo*	Sapindaceae		7	*Cladosporium cladosporioides*	GQ458030.1	98
				7	*Colletotrichum gloeosporioides*	AY266378.1	99
				7	*Fusarium phaseoli*	MH855640.1	98.9
				4	*Ceratobasidium* sp.	KJ471494.1	95
CC6	*Antennaria parvifolia*	Asteraceae	99	8	*Chaetomium globosum*	KM873624.1	91
				6	*Ceratobasidium* sp.	KR259886.1	99
				5	*Cladosporium* sp.	GU797141.1	99
				5	*Fusarium oxysporum*	KJ854902.1	99
CC7	*Erigeron glabellus*	Asteraceae	100	7	*Ceratobasidium* sp.	KC782943.1	98
				6	*Colletotrichum gloeosporioides*	KJ957791.1	99
				6	*Exserohilum* sp.	HQ909080.1	97
				6	*Fusarium oxysporum*	KJ562372.1	99
CC8	*Oxydendrum arboreum*	Ericaceae	100	9	*Didymella* sp.	DQ092504.1	90
				7	*Fusarium oxysporum*	KJ562372.1	98
				6	*Cryptococcus rajasthanensis*	FR870473.1	99
CC9	*Illicium floridanum*	Schisandraceae	100	4	*Fusarium oxysporum*	KJ854902.1	99
CC10	Undetermined	Undetermined	N/A	8	*Penicillium chrysogenum*	MK881028.1.1	93.37
				3	*Penicillium* sp.	HQ130685.1	99
				3	*Colletotrichum* sp.	HM535385.1	93

**Table 3:** Analysis of macronutrients (N, P, and K), pH and total dissolved salts (TDS) of the soil surrounding collected plants from Sumter and Clarke counties.

Plant Sample	N	P	K	pH	TDS (PPT)
**Sumter County plant samples**					
SC 1	Low	High	High	8.20	40.9
SC 2	Low	Low	High	8.57	43.5
SC 3	Low	Low	High	8.63	40.0
SC 4	Low	Low	High	8.76	36.6
SC 5	Low	Medium	High	8.58	44.4
SC 6	Low	Low	High	8.30	45.9
SC 7	Low	High	High	8.19	89.9
SC 8	Low	Medium	High	7.86	107.5
SC 9	Low	Medium	High	8.00	55.3
SC 10	Low	Medium	High	8.45	43.1
**Clarke County plant samples**					
CC 1	Low	High	High	6.93	428.7
CC 2	Low	High	High	7.97	568.3
CC 3	Low	Medium	High	6.87	666.8
CC 4	Low	Medium	High	6.87	403.7
CC 5	Low	Low	High	6.58	712.4
CC 6	Low	Low	High	6.84	96.2
CC 7	Low	Low	Medium	6.74	15.4
CC 8	Low	Low	High	6.84	87.1
CC 9	Low	High	High	6.87	51.6
CC 10	Low	Medium	High	6.45	55.6
